# Genomic Diversity and Structure of *Copaifera langsdorffii* Populations from a Transition Zone Between the Atlantic Forest and the Brazilian Savanna

**DOI:** 10.3390/plants14182858

**Published:** 2025-09-13

**Authors:** Marcos Vínicius Bohrer Monteiro Siqueira, Juliana Sanchez Carlos, Wilson Orcini, Miklos Maximiliano Bajay, Karina Martins, Arthur Tavares de Oliveira Melo, Elizabeth Ann Veasey, Evandro Vagner Tambarussi, Enéas Ricardo Konzen

**Affiliations:** 1 Universidade do Estado de Minas Gerais, Avenida Escócia, 1001, Cidade das Águas, Frutal 38202-436, MG, Brazil; 2 Universidade do Sagrado Coração, Rua Irmã Arminda, 10-50—Jardim Brasil, Bauru 17011-160, SP, Brazil; jusanchez93@gmail.com (J.S.C.); wilson.orcini@gmail.com (W.O.); 3 Centro de Educação Superior da Região Sul, Universidade do Estado de Santa Catarina, Rua Cel. Fernandes Martins, 270, Progresso, Laguna 88790-000, SC, Brazil; mmbajay@gmail.com; 4 Departmento de Biologia, Universidade Federal de São Carlos, Rod. João Leme dos Santos, km 110-SP-264, Bairro do Itinga, Sorocaba 18052-780, SP, Brazil; karimartins@yahoo.com; 5 Inova Genética LTDA, Fazenda Santa Barbara, Rodovia BR 251, KM 18, Cristalina 73850-000, GO, Brazil; arthurmelobio@gmail.com; 6 Departamento de Genética, Universidade de São Paulo (USP), Av. Pádua Dias 11, Piracicaba 13418-900, SP, Brazil; eann.veasey@gmail.com; 7 Departamento de Produção Vegetal, Universidade Estadual Paulista “Júlio de Mesquita Filho”, Av. Universitária 3780, Botucatu 18610-034, SP, Brazil; evandro.tambarussi@unesp.br; 8 Centro de Estudos Costeiros, Limnológicos e Marinhos, Campus Litoral Norte, Universidade Federal do Rio Grande do Sul, Av. Tramandaí, 976, Imbé 95625-000, RS, Brazil; erkonzen@gmail.com

**Keywords:** Atlantic rainforest, Brazilian trees, Cerrado, population structure, SNP markers

## Abstract

*Copaifera langsdorffii* is a neotropical tree widely distributed in the Brazilian Atlantic Forest and Brazilian Savanna. Population genetic analyses can identify the scale at which tree species are impacted by human activities and provide useful demographic information for management and conservation. Using a Restriction site Associated DNA Sequencing approach, we assessed the genomic variability of six *C. langsdorffii* population relicts in a transition zone between the Seasonal Atlantic Forest and Savanna biomes in Southeastern Brazil. We identified 2797 high-confidence SNP markers from six remnant populations, with 10 to 29 individuals perpopulation, in a transition zone between the Seasonal Atlantic Forest and Savanna biomes in Southeastern Brazil. Observed heterozygosity values (0.197) were lower than expected heterozygosity (0.264) in all populations, indicating an excess of homozygotes. Differentiation among populations (*F*_ST_) was low (0.023), but significant (0.007–0.044, c.i. 95%). A clear correlation was observed between geographic versus genetic distances, suggesting a pattern of isolation by distance. Bayesian inferences of population structure detected partial structuring due to the transition between the Atlantic Forest and the Brazilian Savanna, also suggested by spatial interpolation of ancestry coefficients. Through the analysis of *F*_ST_ outliers, 28 candidates for selection have been identified and may be associated with adaptation to these different phytophysiognomies. We conclude that the genetic variation found in these populations can be exploited in programs for the genetic conservation of the species.

## 1. Introduction

In the past 20 years, remarkable progress has been made towards understanding how the loss of biodiversity affects the functioning of ecosystems as well as human populations [1]. These losses have further implications as they can intensify collapses of ecosystem functions. The most recognized and potentially well-documented examples of biodiversity and ecosystem loss have been in the tropics, home to half of the world’s species [2]. Biodiversity loss is largely driven by human activities such as hunting and habitat loss through deforestation, agricultural expansion, industrialization, and urbanization [3].

The Atlantic Rainforest, one of the world’s major hotspots of biodiversity, originally covering approximately 1,500,000 km^2^ from regions of eastern Brazil suffered a large loss of primary habitat due to continuous anthropogenic pressures and now is reduced to 11–16% of its original area, mostly as small and degraded remnants embedded in matrices dominated by pastures, agricultural lands, and urban areas [4]. The Brazilian savanna (Cerrado) has been the target of high human pressures in the last 50 years, leading to an unprecedented loss of biodiversity and deforestation rates [5,6]. It is the most bio-diverse and threatened savannah on the planet, with 48.2% of its original floral cover already lost, and it is being affected by an intense process of habitat fragmentation [7]. It includes a range of vegetation types, such as forests, savannahs, and grasslands, that together reach 12 thousand plant species, of which 44% are endemic [8].

The Neotropical forest-savannah, including Atlantic Rainforest and Cerrado biomes, shows a geographical discontinuity, along with specific soil and climate characteristics, frequently resulting in high levels of genetic structure between populations of endemic biota found on different forest physiognomies [9]. Those transition zones require appropriate conservation efforts [9]. Cerrado, with more than 200 million ha, shares ecological transition zones with four of the five Brazilian biomes: the Amazon (rainforest), Caatinga (semi-arid region), Pantanal (wetland), and Atlantic Forest (coastal forest) [10]. Cerrado and the Atlantic Forest are connected by a Seasonally Dry Tropical Forest (SDTF) in southeastern Brazil. SDTF is among the most devastated forest types because of its strategic location: low slopes and not far from the coastal zone, where agriculture and urban growth easily occur [11]. In addition, the SDTF is second among the most threatened centers of endemism in the Atlantic Forest, with only 7.1% of its original cover remaining and 6.8% of its remaining forest under protection [4,5,6,7,8,9,10,11,12].

Population genetics analysis, through molecular technologies, has been pivotal in understanding the diversity and vulnerability of many forest areas, as well as contributing to the development of conservation strategies [13,14]. A recent meta-analysis at global scale reveals that genetic diversity conservation needs urgent attention from conservationists [15], so more studies on neglected species need to be pursued and effective actions need to be designed after the studies. Recent advances in genomic methods, including next-generation sequencing, whole genome scans and gene-expression pattern analysis, allowed stepping up from a limited number of neutral markers to genome-wide estimates of functional genetic variation [16]. Restriction-site associated DNA sequencing (RADSeq) is a method [17] that enables the identification of several molecular markers, such as single-nucleotide polymorphisms (SNPs), even without a reference genome [18]. By selecting a certain number of RADSeq loci and based on the choice of different restriction enzymes, a large-scale SNP genotyping matrix can be obtained for a target species [18,19]. Thus, a more detailed understanding of population genomics is possible, allowing the development of effective strategies for the conservation of genetic resources [18].

Although genetic analyses have become an integral part of conservation biology, very few conservation genetics studies have been completely integrated into the development of conservation and management strategies and formal policies [20]. For example, much of the genetic diversity of native Brazilian flora has not yet been studied [21], showing a large gap between the supply of plants and the few researchers assigned to them. Therefore, many more studies need to be conducted to appropriately characterize and provide a panorama of the genetic diversity of the species whose environments of origin are the tropics. *Copaifera langsdorffii* Desf., a key and widely distributed species in Atlantic Forest and Cerrado areas in Brazil [22], has been receiving special attention due to its medicinal properties, used not only in traditional, but also in modern medicine. Studies with *C. langsdorffii* to date have been performed with isozymes and microsatellite markers (SSR) [22,23,24,25,26,27,28], detecting patterns of diversity of natural populations, genetic structure of populations in fragmented areas, mating system and gene flow. These previous studies revealed important aspects of neutral genetic diversity but relied on a few molecular markers. In the present study, we moved forward to use a genomic approach based on reduced genomic representation to investigate patterns of adaptive genomic variation in *C. langsdorffii* in a transition zone between Atlantic Forest and Cerrado in Southeastern Brazil. 

Using SNP markers detected through RADseq, we assessed the genetic variability and potential to conserve the genetic diversity of *C. langsdorffii* populations in two different biomes: the Brazilian Atlantic Forest and the Savanna of the Central-West Region of São Paulo state. Our specific goals were to: (1) characterize genetic diversity and population structure of *C. langsdorfii* in Brazilian Atlantic Forest, Cerrado and transition zones, and (2) identify markers under directional selection to assess if transition zones display any response to environmental stressors in populations from distinct phytophysiognomies.

## 2. Materials and Methods

### 2.1. Study Species

*Copaifera langsdorffii*, popularly known as Copaíba (Supplementary Material S2—ESM2), belongs to the Fabaceae family and Detarioideae subfamily [29]. According to [30], *Copaifera* has 72 described species, 16 of which are found exclusively in Brazil [31], and is distributed from the northern to the southernmost regions of the country [32]. The distribution of the species encompasses biomes such as the Amazon Rainforest, Atlantic Forest, and the riparian forests of the Cerrado region [22]. This tropical tree can reach 40 m in height and live up to 400 years. It is pollinated by small insects (*Apis mellifera*, *Trigona* sp., among others), and is predominantly outcrossed [32,33]. The fruits are a follicle type with a black seed surrounded by an orange aryl [31,34], traits that indicate that seed dispersal occurs mainly through animals, especially birds [34].

Natural populations have been gradually eliminated due to habitat degradation and habitat fragmentation. Currently, most populations are restricted to small remnants within forest fragments [25]. *Copaifera* sp. produces an oil-resin that is well described in the literature and may vary in quantity and quality among species [35]. Products derived from *Copaifera* spp. (copaíba oil-resin) are typically extracted via tapping into the trunk rather than logging, and are widely used in medicinal, cosmetic, and industrial applications. Although extraction does not entail felling, traditional and modern practices have been documented to be unsustainable when performed intensively over time, especially without long-term management protocols. Ethnobiological studies in Brazil report that historical extractivist economies were often destructive, even when extraction methods were ritualized or respectful [36]. The oil can be used to treat ulcers, sinusitis, inflammation of the skin, kidneys, and throat. It is also used in the perfume industry for its emollient properties, such as bactericidal and anti-inflammatory, in the manufacture of soaps, bath creams and foams, shampoos, conditioner creams, moisturizing and hair lotions [31,37]. Copaiba plays key ecological functions by contributing to forest structure with high phenotypic plasticity [38], supporting biodiversity through pollination and seed dispersal interactions [39], and enhancing ecosystem services such as carbon storage and soil protection [40].

### 2.2. Sampling Sites

**Figure 1:**
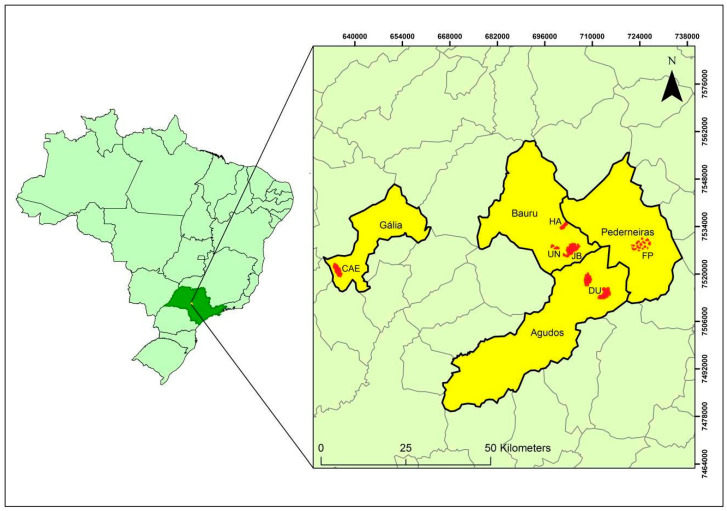
Map of Brazil, highlighting the state of São Paulo (dark green in left map) and the Central-West Region, showing the municipalities and collection sites (in yellow) of *Copaifera langsdorffii* populations: Bauru—JB, UN, HA; Pederneiras—FP, Agudos—DU and Gália—CAE.

The study was conducted in the Central-West Region of São Paulo State, southeastern Brazil, in areas of Cerradão (a forest phytophysiognomy of Brazilian Savannah) and Semideciduous Seasonal Forest (an Atlantic Forest phytophysiognomy). These areas have a Cwa climate (humid temperate climate with dry winter), where summer is hot, with the air temperatures in the hottest month rising above 22 °C; the average rainfall is less than 60 mm in at least one of the months of the season [41]. The sampled areas involved secondary, regenerating, and ecotone forests, and six remaining fragments were selected and defined as populations, located in four municipalities, comprising public and private areas. A total of 95 adult trees were sampled. Population sampling took place between May and June 2015. During sample collection, reproductive individuals were prioritized, and a minimum distance of 10 m between them was maintained. In the municipality of Bauru, three population remnants were sampled: 1. Municipal Botanical Garden of Bauru (JB) with 321.71 hectares (ha) of extension (29 individuals sampled); 2. Campus of the São Paulo State University (UN) with 132 ha (20 individuals); and 3. Aimorés Forest (HA) with 5424.15 ha (11 individuals). In the municipality of Pederneiras, we sampled one population with 14 individuals at the State Forest (FP) with 430 ha. In the municipality of Agudos, a population was sampled with 11 individuals at the reserve of a private property (DU) with 2000 ha. In the municipality of Gália, 10 individuals were sampled at the Caetetus Ecological Station (CAE), with 2178.84 ha (Figure 1). The JB, UN, and FP fragments belong to areas where Savanna and Semideciduous Seasonal Forest vegetation occur, being important ecotones that were considered in this study. The other sampled areas (HA, DU, and CAE) comprise only the composition of Seasonal Semideciduous Forest, and DU stands out for being an area of natural regeneration. For details of *C. langsdorffii* populations sample collection, please refer to Supplementary Material (ESM1 and ESM2), which shows details on their location.

Leaf samples of each individual of *C. langsdorffii* were immediately packed in liquid nitrogen and then stored at −80 °C until DNA extraction. Measurements on tree height, diameter at breast height (DBH), MSL, and geographical coordinates were also gathered during sampling (Supplementary Material).

### 2.3. DNA Isolation and RADSeq Library Preparation

Total genomic DNA was extracted according to the protocol established by the DNeasy Plant Mini Kit manufacturer (QIAGEN, Hilden, Germany), generating high-quality samples according to quantification performed on NanoDrop (Spectrophotometer 2000, Thermo Scientific; Waltham, MA, USA) and 1.2% agarose gel, stained with ethidium bromide.

The sequencing was performed by SNPsaurus LLC (Eugene, OR, USA) using RADSeq [42] and genomic DNA was transformed into RADSeq-like libraries using selective primers. Genomic DNA was first fragmented with Nextera reagent (Illumina, Inc., San Diego, CA, USA), to which small adapters were attached at the end of each fragment. The DNA fragments were then amplified with one of the primers, combining the adapter and extending nine arbitrary nucleotides in genomic DNA with the selective sequence. Thus, only fragments starting with a sequence that can be hybridized by the selective primer sequence have been efficiently amplified. The resulting fragments were fixed at the selective end, presenting random lengths depending on the initial Nextera fragmentation. In this sense, DNA amplified from a particular locus may appear in fragments of different sizes, so careful selection of the library size is not necessary. The RADSeq libraries were sequenced on a HiSeq 4000 (University of Oregon) using a custom sequencing primer that includes the selective sequence so that the entire read is used for genotyping.

### 2.4. SNP Calling and Genotyping

The SNPs were called using custom scripts that created a *de novo* reference from abundant reads and then mapped all the reads to the reference with an alignment identity threshold of 95% [42,43]. Genotype calling was performed using Samtools [44] and bcftools [45] and filtered using Plink 1.9 [46].

Data were filtered excluding non-polymorphic SNP markers or those with more than 10% missing data and SNP loci with minor allele frequencies (MAF) of less than 1%. We also excluded samples with more than 25% missing data. In total, 95 individuals remained for population genomic analyses.

### 2.5. Data Analysis

#### 2.5.1. Genetic Diversity and Structure

Observed (*H*_O_) and expected (*H*_E_) heterozygosities were estimated using both ‘*hierfstat*’ [47] and ‘*adegenet*’ [48] packages in R [49]. *F* statistics, allelic richness, and the respective confidence intervals, based on 1000 bootstrap resamples, were estimated using the R ‘*diversity’* package version 1.9.90 [50].

A matrix of Nei’s (1972) [51] genetic distances was generated and used for constructing a dendrogram through the unweighted pair group method with arithmetic mean (UPGMA) approach [52], using ‘*poppr*’ [53]. The consistency of the nodes was estimated through 1000 bootstrap replications.

Genetic structure was analyzed with a Bayesian approach using Structure v. 2.3.3 [54] under the mixture model, delimiting *K* groups from 1 to 10 with 10 replicates, a 200,000 burn-in period followed by 500,000 MCMC iterations. *K* was chosen according to the Δ*K* method of [55] using Structure Harvester v. 0.6.7 [56]. The genetic structure of populations was also assessed using discriminant analysis of principal components (dAPC) with ‘*adegenet*’ in R [47] and principal component analysis (PCA) with ‘*ape*’ [57], ‘*ade4*’ [58], and ‘*ggplot2*’ [59] packages in R. The coancestry coefficients were also calculated from ‘*tess3r*’, using the *tess3* function [60]. Spatial interpolation of the coancestry coefficients was computed and displayed with ‘tess3r’, along with ‘*raster*’ [61] and ‘*rworldmap*’ [62] packages from R.

#### 2.5.2. *F*_ST_ Outlier Analysis

The discovery of the candidate sites for the divergent selection (*F*_ST_ outliers) was conducted with the FDIST2 method [63], in which the outliers are the *F*_ST_ estimates that lie above the 99% threshold of a simulated distribution of neutral *F*_ST_ values. This method was implemented in the Lositan software v1.0 [64], and the statistical significance of *F*_ST_ outliers was considered using the false discovery rate (FDR) strategy to correct for multiple tests [65].

Estimates of genetic diversity parameters were compared between the total set of markers and the non-neutral loci to estimate the effect of potential adaptive loci on populations from the Semideciduous Seasonal Forest and the savanna environments.

## 3. Results

### 3.1. Genetic Diversity and Structure

The molecular characterization resulted in 2797 SNP markers, most of which had observed heterozygosity values (*H_O_*) lower than the expected heterozygosity values (*H_E_*) in all six fragment populations (Table 1). Genetic diversity indices revealed slight differences among all populations; however, the JB population showed the highest values for all the genetic statistics, followed by FP. The inbreeding coefficients (*F_IS_*) were all significant with an average of 0.209 (0.158–0.251, confidence interval at 95%) (Table 1).

**Figure 2:**
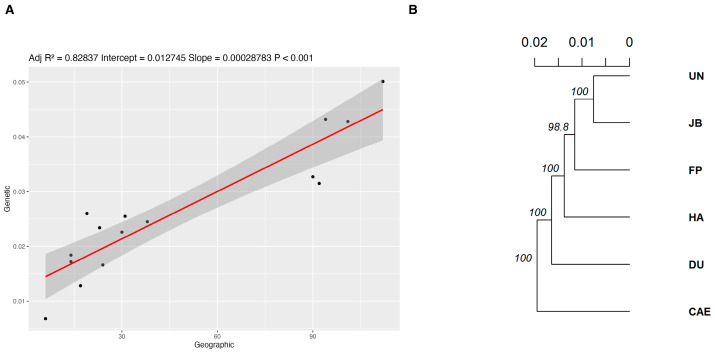
(**A**) UPGMA dendrogram obtained by UPGMA method for six populations of *Copaifera langsdorffii* from the Central-West Region of São Paulo state, southeastern Brazil. The scale indicates Nei’s distance (1972) values and node values are the supporting bootstraps. (**B**) Linear model fitting Nei’s genetic distances with the corresponding geographic distances. The correlation coefficient associated with the model is *r* = 0.9101.

Population differentiation was low (*F_ST_* = 0.023), but significant (0.007–0.044, c.i 95%). Pairwise *F_ST_* suggested a pattern of isolation by distance, with the most isolated population (CAE, approximately 90–112 km away from the other populations) showing higher and significant *F_ST_* estimates (Table 2). The hierarchy of the genotypes in the UPGMA dendrogram (Figure 2A) and the high correlation between the genetic distance and the geographic distance, as inferred from a linear model (Figure 2B), corroborate the tendency of isolation by distance found in pairwise *F_ST_* (Table 2) data.

**Figure 3:**
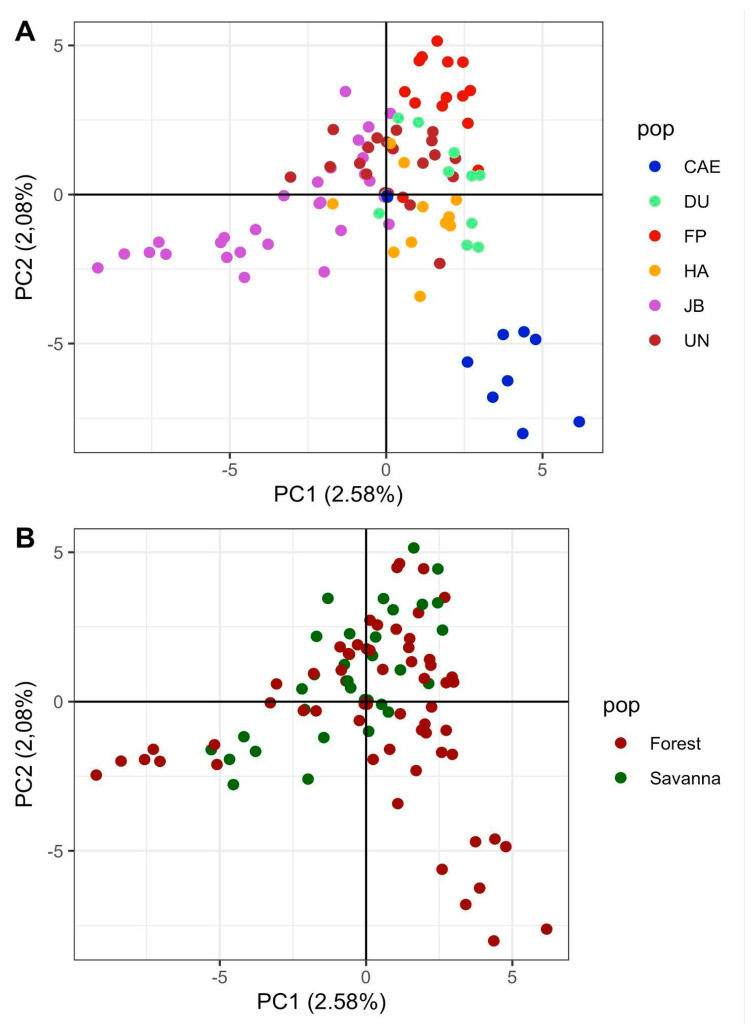
Principal component analysis of SNP markers obtained from *Copaifera langsdorffii* individuals sampled from sites within the Seasonal Semideciduous Forest and Brazilian savanna (Cerrado) at different collection sites. (**A**) Scatter plot colored by forest type. (**B**) Scatter plot colored based on collection site.

Through principal component analysis, the two first components explained only 4.66% of the total genetic variability found in 95 genotypes (Figure 3). The samples from CAE and JB were more dispersed from the others (Figure 3A). The individuals collected in areas of Seasonal Semideciduous Forest (in the graph marked as Forest) and the individuals collected in the Savanna area (in the graph marked as Savanna), were marked by different colors (Figure 3B). However, a partial overlap was observed between the two groups.

**Figure 4:**
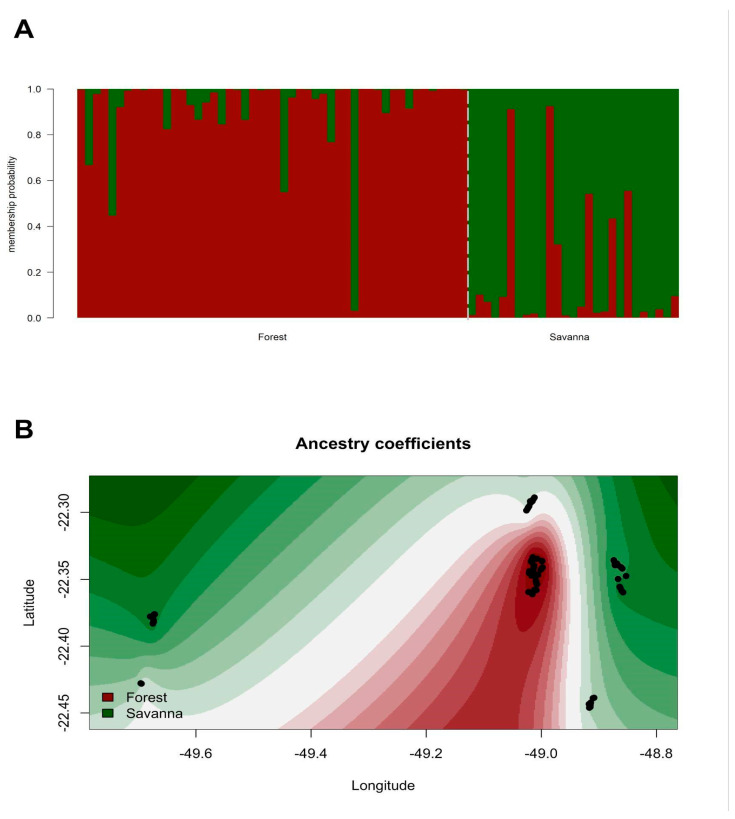
Genetic structure of *Copaifera langsdorffii* sampled from sites with Seasonal Semideciduous Forest and Brazilian savanna (Cerrado). (**A**) Genetic structure display based on K = 2 obtained with Structure. (**B**) Spatial interpolation of ancestry coefficients as obtained with Tess.

Despite their overlap, a trend of separation between forest and savanna ecosystems can be observed, for example, in JB, where samples belonging to the forest are mostly separated from the savanna (compare graphs A and B in Figure 4). The isolation of distance of CAE, however, is also clear from the comparison (Figure 3A). Given those clues, further Bayesian analysis showed the highest ΔK estimation in a population structure of K = 2, with most of group I (red) belonging to the genotypes collected in the Forest ecosystem, while most of group II (green) included the genotypes collected in the Savanna (Figure 4A). This pattern, however, was not clear, as a mixture of ancestry between both groups was also observed. After spatial interpolation of the ancestry coefficients, using TESS structure analysis, an overall trend of separation between Savanna and Forest was also detected (Figure 4B). Once more, the site of CAE shows a genetic separation even from the other samples of the Forest group, suggesting more genetic divergence from the other sites.

### 3.2. Outlier F_ST_ Analysis

**Figure 5:**
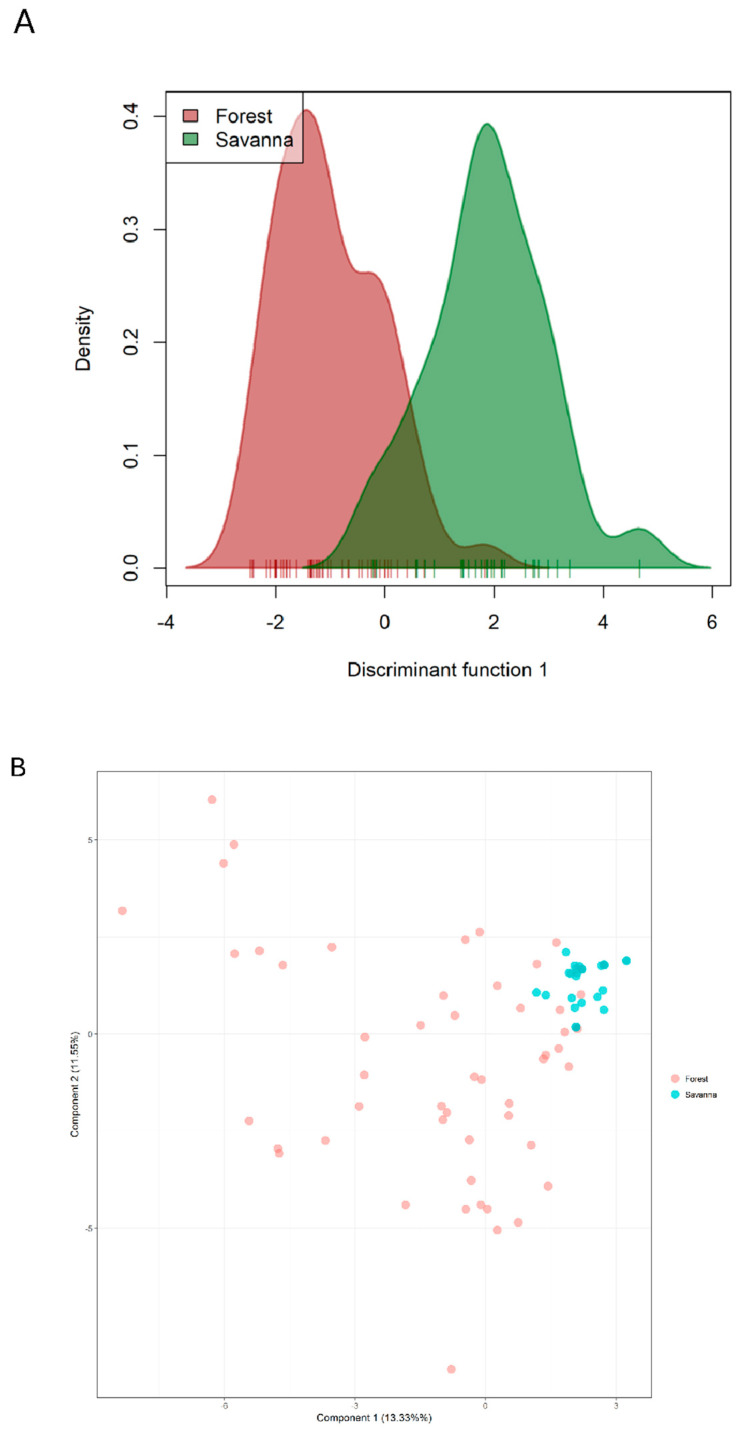
dAPC clustering of outlier SNP loci as determined based on the genotyping-by-sequencing of *Copaifera langsdorffii* populations sampled from two forest types: Season semideciduous forest and Brazilian savanna (Cerrado). (**A**) Individual density graph according to the defined groups (Cluster 1—Seasonal Semideciduous Forest; and Cluster 2—Savanna), along the first discriminant function obtained by DAPC, with cutoff = 0.001. (**B**) Scatter plot based on 28 outlier loci collected from six fragments in the Central-West Region of São Paulo State, Brazil.

Twenty-eight outlier loci were detected with Fdist2 analysis, which were significant after the FDR correction (Supplementary Material S1). All were confirmed as the most significant loci to separate the two groups by dAPC. Of these loci, 21 are monomorphic in the Savanna samples, indicating that they may be relevant in adapting the species to this environment. The dAPC analysis discriminated the same 28 loci as the SNPs with the greatest contribution to the differentiation of the clusters. The density graph (Figure 5A) shows that the samples are better grouped according to this contrast of environments using the 28 outlier loci. This dispersion pattern is very evident in the dAPC scatter plot (Figure 5B).

**Figure 6:**
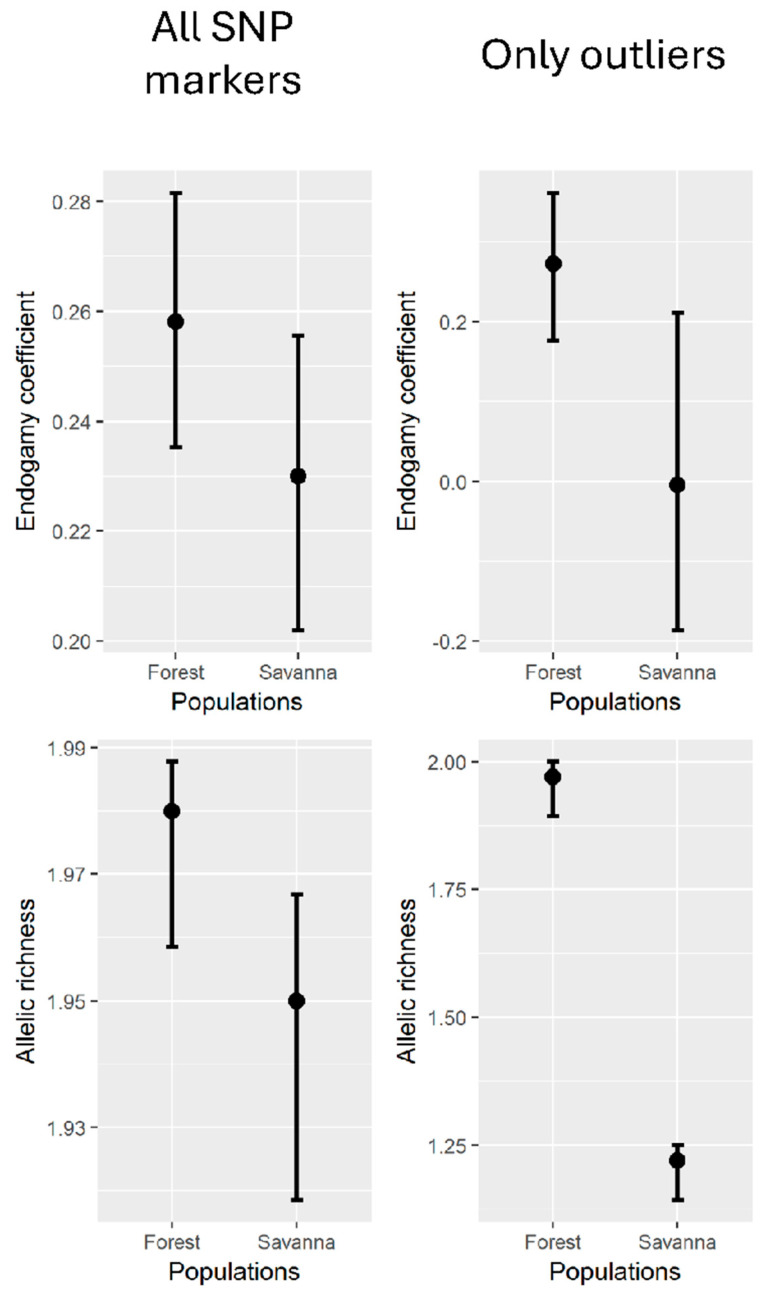
Inbreeding coefficient and allelic richness based on SNP markers obtained after genotyping-by-sequencing with samples of *Copaifera langsdorffii* collected from two forest types: seasonal semideciduous forest and Cerrado. The left column shows estimates with all SNP loci, while the right graph estimates are based on the outlier loci only. Vertical lines stand for ±95% CI.

From our previous classification into two groups of ancestry, the Forest and Savanna groups, no clear differences in the genetic diversity estimates were observed (Figure 6). Here, we also estimated the allelic richness between the two groups, revealing that the coefficient of inbreeding is lower, on average, in the Savanna than in the Forest group. The allelic richness is also lower in the Savanna, on average. That difference is not clear, however, from the analysis of all SNP markers. When only the outliers are compared for the estimates, the inbreeding is indeed lower in the Savanna, and the allelic richness is significantly lower in the Savanna than in the Forest group (Figure 6).

## 4. Discussion

Our study revealed that all the sampled sites had an excess of homozygotes, whether belonging to the Forest or the Savanna group. Moreover, an important result revealed by our genotyping and sampling is a weak but noticeable genetic structure between the Forest and Savanna groups. Despite the spatial structure due to the isolation by distance, a trend of structuring by ancestry between the two groups was detected based on Bayesian inferences. Population structuring between Forest and Savanna was more prominent based on 28 outlier loci that were detected from the overall set of SNP markers, 21 of them in homozygosity in the samples belonging to the Savanna.

These 21 monomorphic loci in Savanna populations were identified based on the results of the *FST* outlier analysis. Using FDIST2 and confirmed by DAPC (Discriminant Analysis of Principal Components), we identified 28 candidate loci putatively under selection. Of these, 21 loci were found to be fixed (monomorphic) in the Savanna populations while remaining polymorphic in Forest populations.

This pattern suggests that selective pressures specific to the Savanna environment, such as nutrient-poor soils, water stress, and recurrent fire regimes, may have favored certain alleles, leading to the loss of polymorphism at these loci. In population genomics, loci that deviate significantly from neutral expectations (outliers) and show habitat-specific fixation are widely considered candidates for local adaptation, as they likely reflect directional or purifying selection eliminating maladaptive variants [23,63,66,67].

Similar approaches in forest trees have demonstrated that outlier SNPs fixed in contrasting environments often carry signatures of adaptive divergence [68,69]. Therefore, our interpretation is consistent with the broader framework of evolutionary and landscape genomics, where fixed outlier loci are considered indicative of adaptation to local edaphoclimatic conditions.

### 4.1. Excess of Homozygotes in All Sites

The excessive levels of homozygotes detected in each site are in line with most studies performed with different types of molecular markers, such as isozymes [28] and microsatellites [22,26,27,39,70]. The first recorded genetic study with natural populations of *C. langsdorffii* was that of [71], who identified an excess of homozygotes in two riparian areas based on allozyme markers. Anthropic intervention over the years may have directly affected the dispersers of the species and, consequently, generated an increase in homozygotes among these populations. Ref. [28] found, based on isoenzymatic markers, that 60 *C. langsdorffii* individuals in three populations (Cerrado, Atlantic Forest and Riparian Zone) had high observed and expected heterozygosity when compared to most species, both tropical and temperate. According to the authors, the Savanna and Semidecidual Seasonal Forest populations expressed a tendency for an excess of homozygotes. Exceptionally, they were more heterozygous in the Riparian Zone, contrary to what is commonly observed for other vegetation formations. Similar data were obtained by [26] analyzing 80 *C. langsdorffii* individuals based on six SSR loci, finding *H_o_* = 0.715 and *H_e_* = 0.882, on average, for the three forest areas collected, also indicating an excess of homozygotes. An excess of homozygosity suggests inbreeding, the presence of null alleles, or the occurrence of the Wahlund effect. As *C. langsdorffii* has hermaphroditic flowers, the excessive homozygosity could be explained by selfing and mating among relatives [70].

### 4.2. Outcrossing Rates May Be High, but There Is Also Isolation by Distance

Despite the current excess of homozygotes, the *F*_ST_ values observed in the sampled sites of *C. langsdorffii* suggest high levels of outcrossing [72], which may result from historical gene flow among sites on a geological timescale. A similar study with *Copaifera reticulata* Ducke, in the Amazon region, using SSR markers, concluded that most of the genetic variation was found within the collection areas, with weak genetic differentiation between areas [73]. Similar data were observed with the tropical species *Protium spruceanum* Benth Engl [74] and *Prunus africana* (Hook. f.) [75], indicating low population genetic structure for both. Low *F*_ST_ values are found for most tree species (Hamrick and Godt 1996) [76]. This can be explained by a combination of factors throughout the life of these plants, specifically their breeding system [77]. Ref. [78] pointed out that the reasons for these low *F*_ST_ values may be because tropical trees have low but significant rates of self-fertilization and bi-parental crossing, as well as genetic drift due to the low population densities of most species.

A strong association between genetic divergence and geographic distances was observed for all six sites. Based on the environmental landscape and in a seed dispersal study [70] with the same species in the same area, four sampled sites are completely isolated from each other (HA, CAE, FP, and DU) (Figure 1). Even with the sites JB and UN considered as two distinct populations, there is evidence that in the recent past, they were a unique large forest fragment. Ref. [27] also observed a strong association between genetic and geographic distances for *C. langsdorffii* in two experimental stations from the Forest Institute (Assis and Itirapina), located at Pedregulho Conservation Unit and in a private property at Brotas municipality, both in São Paulo State.

Spatial Genetic Structure (SGS) analysis for the *C. langsdorffii* populations suggested isolation by distance. Similar results were found in previous studies [22,27], indicating that spatial isolation is a product of forest fragmentation, which decreases the effective population size and consequently the genetic diversity. Ref. [70] highlighted seeds of *C. langsdorffii* generally have short-distance dispersal, and the isolation by distance seems due to restricted pollen and seed flow among populations. The SGS characterization, along with effective population size and effective pollen donor size estimations and models of both pollen and seed dispersals, are fundamental parameters to be considered to set up efficient strategies of tree conservation.

### 4.3. Population Structure Reflects the Transition Between Forests and Savannas

The Bayesian hierarchical clustering analysis showed that the six sampled sites were structured into two larger groups (K = 2), suggesting a distinction between the genotypes belonging to the Savanna and the Semideciduous Seasonal Forest. The spatial interpolation of the ancestry coefficients also revealed a trend of separation between the Savanna and the Forest. Because the Bauru region is an important transition area [79], the analysis of genetic structure corroborates the initial idea that there is a transition between these two forest formations. On the one hand, we observed two distinct vegetation structures on the JB population, illustrating here an important ecotone, and certainly this disposition is manifested by the separation of its genotypes (Figure 3 and Figure 4). In addition, the DU population, whose forest area is a continuous part of the JB Semideciduous Seasonal Forest (Figure 3 and Figure 4), presents its genotypes isolated from most of the JB genotypes sampled in Savanna areas. According to [80,81], stretches today covered by the Savanna vegetation correspond to areas where savannahs and/or countryside physiognomies predominated in the past, according to the descriptions of [82], in a study on the native vegetation of Bauru. According to [81], the comparison between old and current aerial images of the vegetation allows us to verify a change from countryside and savannah physiognomies to forest physiognomy.

### 4.4. Outlier Loci Analysis Highlights Putative Local Adaptations

Out of 28 candidate outlier loci pointed out by the neutrality test, 21 were monomorphic loci, leading to the hypothesis that the fixed loci have important evolutionary significance in the study areas. One hypothesis is that most of these loci may be associated with important alleles for adaptation to the savanna, and genotypes with polymorphism in such regions are negatively selected. It is likely that part of the 21 outlier loci that were fixed in these areas may be related to the adaptation to the edaphoclimatic conditions found in that environment.

One of the goals of evolutionary biology studies is to determine the influences of positive and purifying selection as well as neutral forces that shape the landscape genetic variation and natural populations that span large climate gradients, offering an ideal opportunity to investigate these evolutionary forces [23,67]. However, population genomic studies with tropical trees aiming to understand the speciation processes are still incipient [83], specifically in transition areas of vegetative physiognomies. Due to the strong anthropogenic pressures in the region of the present study, understanding adaptive genetic responses to climate change is one of the main challenges for the preservation of local biological diversity. Similar studies have addressed genetic and ecological variables to understand changes in character expression or allelic frequencies over a series of contiguous populations within a given geographical distance.

Recent studies have sought to identify genes associated with geographic gradients, mostly in temperate regions. Meger et al. [68] identified 781 SNPs in 389 phenology-related genes associated with geographic, climatic, and phenotypic gradients in natural populations of *Quercus robur*, providing evidence of local adaptation to current and future climate conditions. Similarly, Kremer et al. [69] identified 38 outlier SNPs through whole-genome screening that showed strong geographic structure, allowing for the discrimination of four Western European white oak species and highlighting signatures of spatial genetic differentiation.

Our results corroborate those observed by [66], who suggest genetic differentiation in *Erythrina falcata* through a small spatial scale analysis, despite the increasingly reduced gene flow. The authors hypothesized that heterogeneous environments may cause molecular divergence, possibly associated with local adaptation, a situation similar to what was observed in the transition area in the Central-West Region of São Paulo, between the two phytophysiognomies studied.

Our findings point to several directions for future research that can strengthen conservation strategies for *C. langsdorffii*. The detection of outlier loci potentially associated with local adaptation highlights the need for functional studies to validate their role under contrasting phytophysiognomies. Landscape genomic approaches that integrate environmental variables may further elucidate adaptive processes and support the definition of seed transfer zones resilient to climate change. In addition, coupling demographic and genomic data will be essential to refine estimates of effective population size and dispersal, improving strategies to maintain genetic connectivity. Finally, the establishment of long-term monitoring and the integration of in situ and ex situ conservation initiatives will be crucial to safeguard both neutral and adaptive components of genetic diversity in fragmented landscapes.

## 5. Conclusions

Our study revealed that all population relicts sampled have an excessive level of homozygotes, which may be due to inbreeding or other underlying factors that need to be addressed for the proper design of conservation practices and management. A clear pattern of isolation by distance was detected from our study, with high correlation between geographic and genetic distances. Therefore, there is spatial genetic structure among the population relicts. Moreover, Bayesian inferences obtained by two algorithms, STRUCTURE and TESS, showed population structure of the ancestry coefficients. Spatial interpolation of the ancestry coefficients supported a trend of separation by the phytophisiognomy, the Atlantic Forest, or the Brazilian Savanna. The transition zone between these biomes partially influences population structuring.

## Figures and Tables

**Table 1:** Genetic diversity estimates for six populations of *Copaifera langsdorffii* in an ecotone region between Atlantic Forest and Brazilian savanna in southeastern Brazil.

Population	*N*	*H*_O_	*H*_E_	*F*_IS_
JB	29	0.205	0.257	0.203 *
UN	20	0.189	0.247	0.235 *
HA	11	0.187	0.244	0.235 *
FP	14	0.203	0.245	0.173 *
DU	11	0.178	0.238	0.251 *
CAE	10	0.196	0.233	0.158 *
Mean	-	0.197	0.264	0.209

*N*: number of samples, *H*_O_: observed heterozygosity, *H*_E_: expected heterozygosity, *F*_IS_: inbreeding coefficient (* *p* < 0.05). JB, UN, HA, FP, DU, and CAE are the acronyms for population names (see methods).

**Table 2:** Pairwise *F_ST_* estimated for *Copaifera langsdorffii* populations (below diagonal) and pairwise geographic distances (km) (above diagonal).

Pop.	JB	UN	HA	FP	DU	CAE
JB	_	6	14	23	19	94
UN	0.0068	_	17	24	14	90
HA	0.0172	0.0128	_	31	30	92
FP	0.0234 *	0.0166	0.0255	_	38	112
DU	0.0260	0.0184	0.0226	0.0245	_	101
CAE	0.0432 *	0.0327 *	0.0315 *	0.0501 *	0.0428	_

* *p* < 0.05.

## Data Availability

The datasets generated during and/or analyzed during the current study are available from the corresponding author on reasonable request.

## Supplementary Materials

The following supporting information can be downloaded at: https://www.mdpi.com/article/10.3390/plants14182858/s1. Supplementary Material S1: Excel spreadsheet; Supplementary Material S2: A—Jardim Botânico (JB), B—UNESP-Bauru (UN), C—Horto Aimorés (HA), D—Floresta Pederneiras (FP), E—Duratex (DU), F—E.E. Caetetus (CA), G—Copaifera langsdorffii leaves, H—Copaifera langsdorffii trunk
