# Supplementary material for: Genomic Diversity and Structure of Copaifera langsdorffii Populations from a Transition Zone Between the Atlantic Forest and the Brazilian Savanna
Source: Plants (Basel). 2025 Sep 13;14(18):2858. doi: 10.3390/plants14182858 (PMC12473662; doi:10.3390/plants14182858)

Supplementary Material 2

A – Jardim Botânico (JB), B – UNESP-Bauru (UN), C - Horto Aimorés (HA), D - Floresta Pederneiras (FP), E - Duratex (DU), F - E.E. Caetetus (CA), G - *Copaifera langsdorffii* leaves, H - *Copaifera langsdorffii* trunk

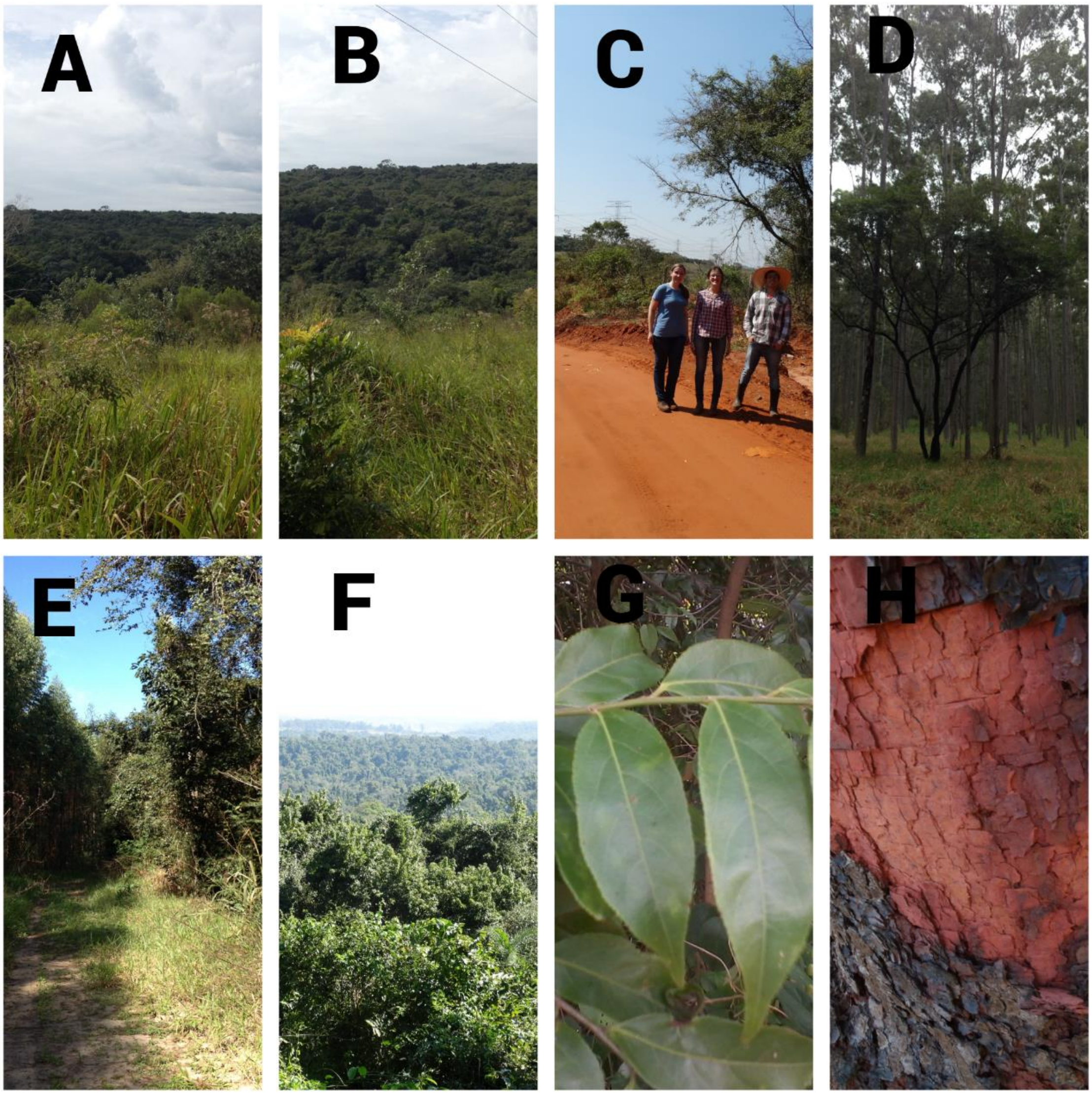

Supplement: Supplementary file 1 [file plants-14-02858-s001.zip › plants-3826667-supplementary S2.pdf]
